# Acupuncturation

**Published:** 1829-07-01

**Authors:** 


### HOTEL DIEU DE CAEN.*

#### Acupuncturation.

In nothing is fashion, omnipotent fashion, more conspicuous than in medicine. A little while ago the town rang with "acupuncture;" every body talked of it; every one was curing incurable diseases with it; but now not a syllable is said upon the subject, and acupuncturation would seem to be quietly consigned to "Lethe's silent stream." In France, however, the advocates of the measure would seem to be as hot as ever, and cases are constantly recorded of the wonderful benefits and cures it has accomplished. In the Archives Générales for last October, two cases are reported from the practice of M. Trouve, where it *seemed* (for alas! in these matters we are frequently sadly taken in) to produce good effects. The first was that of a young woman labouring under many of those strange hysterical symptoms so commonly met with in practice, and that to a very distressing degree. A great variety of general and local means were put in practice with little or no effect, when M. le Médecin en chef resorted to the needles, and employed them assiduously for many days, as often as the slightest premonitory symptom of a hysterical paroxysm made its appearance. The patient soon left the hospital cured. The second patient was also a young woman, who had suffered for seven years from paralysis of the right lower limb following a fall upon the back, and obliging her to go constantly on crutches. Four applications of the needles sufficed to give

---

* Archives Générales, October, 1828.

her perfect use of the long palsied limb, and shortly afterwards this patient also left the hospital.

We suppose the modus operandi of acupuncture, at least in cases of this description, is to be considered as similar to that of incantations, cauls, &c. for it is notorious that many a malady has yielded to the potent spell of some old beldame, which had long resisted the professional skill of the regular descendants of Hippocrates. Whatever the *mode* in which the needles act may be, if they *have* an action, and that a good one, they are worth a trial now and then in those nervous or hysterical disorders, on which scientific measures are completely thrown away. We have seen a most salutary salivation produced by *bread pills*, in a very hypochondriacal patient, who fancied he had syphilis, and that he *ought* to be put under the influence of mercury. The pills of course were said to be mercurial, and particular injunctions were given him to leave them off as soon as the mouth should be affected.

---

XI.

